# Effect of a single vectored thermal pulsation treatment of Meibomian gland dysfunction patients under controlled environmental conditions

**DOI:** 10.1038/s41598-022-20994-y

**Published:** 2022-10-06

**Authors:** Andrea Novo-Diez, Alberto López-Miguel, Itziar Fernández, Marta Blanco-Vázquez, Cristina Valencia-Sandonís, Amalia Enríquez-de-Salamanca, María J. González-García, Margarita Calonge

**Affiliations:** 1 IOBA (Institute of Applied Ophthalmobiology), Universidad de Valladolid, Campus Universitario Miguel Delibes, Paseo de Belén 17, 47011 Valladolid, Spain; 2 Biomedical Research Networking Center in Bioengineering, Biomaterials and Nanomedicine (CIBER-BBN), Valladolid, Spain

**Keywords:** Eye manifestations, Clinical trials

## Abstract

To assess the prophylactic effect of LipiFlow treatment in Meibomian gland dysfunction (MGD) patients exposed to an adverse environmental humidity. MGD patients were exposed to normal (23 °C; 50% relative humidity; 30 min) and adverse (23 °C; 10% relative humidity; 2 h) controlled environments consecutively during baseline and follow-up visits (3, 6, and 12 months) after a single LipiFlow treatment. Ocular Surface Disease Index (OSDI), lipid layer thickness (LLT), fluorescein tear break-up time (TBUT), corneal and conjunctival staining, change in dry eye symptoms questionnaire (CDES-Q), and Meibomian gland yielding liquid secretion (MGYLS), were assessed. Linear mixed-effects and cumulative logit mixed models were fitted to assess the effect of the LipiFlow treatment over time and within the controlled environments. Seventeen females and 4 males (59.6 ± 9.4 years) completed the study. LLT and TBUT did not vary significantly (*p* > 0.05) after LipiFlow treatment. OSDI, corneal and conjunctival staining, and MGYLS scores were improved (*p* ≤ 0.01) 12 months after treatment. After the adverse exposure, corneal staining increased at all visits (*p* = 0.01), and there was no significant improvement in CDES-Q scores after LipiFlow treatment (*p* ≥ 0.07). One LipiFlow treatment improved objective and subjective outcomes in MGD disease for at least one year. Further studies are needed to support that LipiFlow might also help as an adjuvant to avoid acute flares against an adverse environmental humidity.

## Introduction

Meibomian gland (MG) dysfunction (MGD) is the leading cause of ocular surface disease^1^. It is characterized by hyperkeratinization of the ductal epithelium and increased viscosity^2^ of the polar and non-polar lipids of the meibum secretion. These changes result in obstruction of the palpebral Meibomian gland orifices through which, under normal circumstance, the oily meibum secretion is delivered to the ocular surface where it forms an integral component of the tear film. Blockage of meibum secretion may result in alterations in the lacrimal functional unit, such as the tear film, and may cause symptoms of eye irritation, clinical inflammation, and other symptoms that are characteristic of ocular surface disease^2^.

To remove the obstruction and to improve the glandular secretion in MGD patients, lid warming, and mechanical lid hygiene have been traditionally recommended. However, the lack of a standard protocol to perform lid hygiene can result in a low and variable effectiveness^3,4^. Moreover, warm compresses need to be reheated at least every two minutes, which makes it a time consuming procedure^5,6^ that can compromise the patient’s commitment to the treatment and reduce its effectiveness^3,4,7^. New technologies have been developed to facilitate MGD management^8^, such as the vectored thermal pulsation provided by LipiFlow (Johnson & Johnson Vision, Santa Ana, CA, USA). This instrument consists of an eyelid warmer that applies heat at 42 °C on the palpebral surface of both lids, and an eye cup that simultaneously applies pulsating pressure via an inflatable air bladder upon the closed eyes. The melting point of MG secretion varies from 30 to 40 °C and is even higher in obstructive MGD. For this reason, the LipiFlow device reaches 42 °C. The bladder inflates and deflates, massaging the eyelids from the MG ducts to the MG orifices. The combination of heating and massage improves MG secretion. The treatment is performed in the clinic during a single session of 12 minutes^7,9,10^.

A single LipiFlow therapy session improves MG secretion and ocular surface signs, such as increased lipid layer thickness (LLT), reduction in partial blinking, increased tear break-up time (TBUT), and reduction of symptoms for up to 12 months^4,11–14^. Following a single in-office treatment, improvements in MG secretion, TBUT, and ocular symptoms were still present at 9 and 12 months^4,11,12^. Even after the 3 years following a single treatment, MG secretion and improved symptoms were sustained^15^.

The efficacy of LipiFlow therapy has also been demonstrated in Sjögren syndrome patients^16^, patients with ocular surface disease after refractive surgery^17^, and subjects with contact lens discomfort^18^. When comparing LipiFlow efficacy with warm compress and lid hygiene, improved symptoms^7,19–21^, MG secretion, and TBUT has been reported^17,20^. A reduction in the tear evaporation rate also occurs after 4 and 12 weeks compared to heat masks such as the EyeGiene® and the Blephasteam®^22^. However, Tauber et al.^23^ found no difference between LipiFlow and pulsed light treatment. With respect to drug therapy, Hagen et al.^24^ observed a greater improvement with one session of LipiFlow than in 3 months of treatment with doxycycline.

People worldwide are constantly exposed to adverse environments, such as low humidity, high temperature, and/or air flow, especially under indoor conditions. These conditions increase water evaporation and thinning of the tear film^25^. Thus, adverse environments exacerbate dry eye-related signs and symptoms, not only in dry eye (DE) disease patients but also in normal subjects and contact lens wearers^26–29^. Furthermore, MGD is the most frequent cause of the evaporative subtype of DE, although, evaporative and aqueous-tear deficient DE usually coexist^30^.

Controlling the indoor environment increases the measurement reliability of the ocular surface signs involving the tear film because patients are always exposed to the same environmental conditions during the study visits^27,31–34^. This research methodology minimizes potential confounding factors^34^. While numerous studies have shown that LipiFlow therapy is an adequate treatment for MGD management^4,7,9–13,15,35^, no previous study has evaluated the efficacy of LipiFlow therapy under controlled environmental conditions.

Furthermore, previous studies have shown the prophylactic efficacy of several DE therapies to ameliorate the worsening of the ocular surface (acute flare) when patients are exposed to adverse environmental conditions^36–39^. However, the ability of LipiFlow therapy to avoid DE exacerbation when MGD patients undergo adverse environmental humidity during their daily activities has not been studied. Therefore, the first aim of the present study was to assess the efficacy of a single LipiFlow treatment in MGD patients over a 12-month period under normal environmental conditions. The second aim was to evaluate the prophylactic benefits of LipiFlow in MGD patients undergoing an adverse environmental humidity exposure.

## Materials and methods

We conducted a prospective, single-centre, open-label study that was registered at the US National Institutes of Health (ClinicalTrials.gov), ID: NCT03843983. The study protocol was approved by the East Valladolid Health Area Ethics Committee (Valladolid, Spain), and conducted in compliance with the tenets of the Declaration of Helsinki. Participants were recruited from the Institute of Applied Ophthalmobiology (IOBA), University of Valladolid, Spain. Written informed consent was signed by all participants prior to enrolment.

### Patients

Patients with ocular surface disease due to MGD were recruited. Inclusion criteria were age 18 years or older; ocular surface disease index (OSDI) questionnaire ≥ 13^40^; MG yielding liquid secretion (MGYLS) score of the lower lid in both eyes ≤ 12^10^; and at least 10 out of 15 functioning MGs in the lower eyelid^41^. Exclusion criteria were any ocular pathology other than ocular surface disease due to MGD; unwilling to abstain from the use of systemic or topical treatments, including manual lid hygiene (except for ocular lubricants) for MGD or DE for the whole study duration; history of severe ocular inflammation or infection in the previous six months; eyelid abnormalities that affected lid function; any ocular surgery or trauma that may have affected corneal sensitivity and/or normal tear distribution in the previous six months; use of contact lenses 15 days before inclusion and during the study; punctual plugs or punctual occlusion within the past three months; and pregnancy or breastfeeding.

### Study design

**Figure 1:**
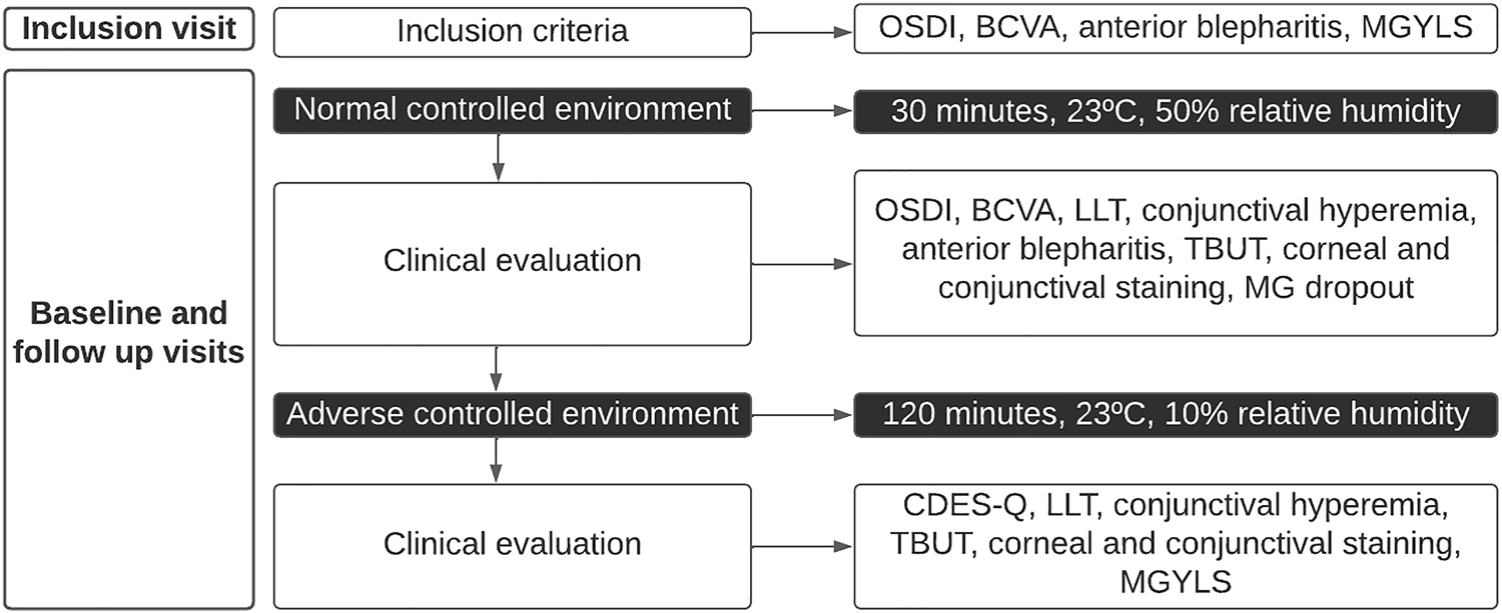
Chronological scheme of the tests performed at each visit. OSDI = ocular surface disease index, BCVA = best corrected visual acuity, MGYLS = meibomian gland yielding liquid secretion, LLT = lipid layer thickness, TBUT = tear break-up time, MG = Meibomian glands, CDES-Q = change in dry eye symptoms questionnaire.

The scheme of the tests performed in the study is summarized in Fig. 1. Patients attended a total of six visits: an inclusion visit, baseline visit, LipiFlow treatment visit, and three follow-up visits scheduled 3, 6, and 12 months after treatment. At the inclusion visit, subjects were recruited after checking inclusion and exclusion criteria. In addition, anterior blepharitis was assessed, and the best corrected visual acuity (BCVA), which served as the safety outcome measure, was determined^42^. Both measures were also assessed in the follow-up visits. If eligible, MGD patients were scheduled for the baseline visit within the next 14 days.

The protocol at the baseline and the 3-, 6- and 12-months follow-up visits was at follows: Participants attended the clinic without any artificial tear use for at least two hours before the visit. Within an environmental chamber (Controlled Environmental Laboratory, CELab)^42^, they were exposed consecutively to a normal and an adverse environment. The first exposure was for 30 min in a normal controlled environment maintained at 23 °C and 50% relative humidity^42^. Following that exposure, the clinical tests were performed within the same environment. Subsequently, patients were exposed for 2 h to an adverse controlled environment: the relative humidity was lowered to 10% (desiccating stress environment) while maintaining the temperature at 23 °C. Following this exposure, the clinical tests were performed again within the same adverse environment.

The single LipiFlow treatment visit was scheduled 2–7 days after the baseline visit. Two drops of topical anaesthetic per eye were instilled followed by placing one LipiFlow Activator on each eye^10^. Patients were asked to close their eyes during the treatment. After 12 min, the Activators were removed by a practitioner^7^. No other treatment or examination followed the removal of the Activators. Each subject was then evaluated at 3-, 6-, and 12-months after the LipiFlow treatment visit to assess variations over time in both the normal and the adverse controlled environment.

### Study parameters

#### Ocular symptoms

Symptoms during the preceding two weeks before each visit were assessed using the OSDI questionnaire^40^. Changes in symptoms after two hours under the adverse controlled environment^29,32,42,43^ were evaluated by the change in DE symptoms questionnaire (CDES-Q)^44^. In this second questionnaire (CDES-Q-1), the patients were asked if they felt better, worse, or the same in comparison to the previous 30 min when they were under the normal controlled environment. If they recorded better or worse feelings, in a final questionnaire (CDES-Q-2) they were asked to score that change in a scale from 0 to 10, where 0 meant slightly better or worse, and 10 meant extremely better or worse^44^.

#### Ocular surface signs

After the 30 min normal environment exposure, in addition to the OSDI questionnaire, clinical tests were performed in the following order: monocular BCVA, the safety outcome measure, was evaluated using the Early Treatment Diabetic Retinopathy Study (ETDRS) (Topcon CO LDT, Tokyo, Japan) chart at 4 m^29^. LLT was measured with the LipiView II interferometer (Johnson & Johnson Vision, Santa Ana, CA, USA). Signs of conjunctival hyperaemia were evaluated based on the Efron grading system with ratings from 0 (no hyperaemia) to 4 (high hyperaemia)^45^. The presence of anterior blepharitis as a result of eyelid redness and swelling was also evaluated using the Efron grading system for contact lens complications, ranging from 0 (no blepharitis) to 4 (high blepharitis)^45^.

Fluorescein TBUT was defined as the time between the last of three blinks and the appearance of the first dry spot. To perform the TBUT test, 5 µl of 2% sodium fluorescein (Bausch & Lomb S.A., Bridgewater, NJ, USA) were instilled with a pipette. The procedure was repeated 3 times to record the mean value^42^.

Fluorescein corneal staining was graded 2 min after the instillation of 5 µl of 2% sodium fluorescein. The Cornea and Contact Lens Research Unit (CCLRU) scale 0 (no staining) to 4 (severe staining) was used, and the total score was recorded after adding the scores of the five corneal areas (maximum score 20)^46^.

Conjunctival staining was assessed using Lissamine green strips (I-DEW green, Entod Research Cell UK Ltd., London, UK) that were wet with 25 µl sodium chloride 0.9%. Staining was graded according to Oxford scheme in the nasal and temporal areas 0 (no staining) to 5 (severe staining). The sum of both area scores was recorded^47^.

Meibography images were obtained using the LipiView II interferometer to quantify the MG percentage dropout, which was calculated by dividing the area with no visible glands by the entire conjunctival area^48,49^. For calculating the MG dropout, a masked observer analysed the images randomly, and quantified the MG dropout area using the Image J software (National Institutes of Health, Bethesda, MD, USA)^49^.

After the 2 h adverse environment exposure, in addition to the CDES-Q, the LLT, conjunctival hyperaemia, TBUT, corneal and conjunctival staining were evaluated again. Finally, to assess MG secretion, the MGYLS score was obtained using the MG evaluator (MGE, Johnson & Johnson Vision, Santa Ana, CA, USA) to ensure measurement consistency. Fifteen MGs of the lower lid margin were evaluated, five in each nasal, central, and temporal region. Expressed secretion characteristics were assessed in each of the 15 glands based on the following grading scale: 3 (clear liquid secretion), 2 (cloudy liquid secretion), 1 (inspissated/toothpaste consistency), and 0 (no secretion). Thus, the total score ranged from 0 to 45^10^.

Finally, to assess the satisfaction that patients experienced with the LipiFlow treatment, they were asked to mark with a vertical line on a 10-mm horizontal visual analogue scale after undergoing the LipiFlow therapy during the treatment visit. The scale ranged from 0 to 10, where 0 meant the lowest satisfaction and 10 the highest.

### Statistical analysis

The calculation of sample size was based on TBUT, considering that a difference of two seconds in TBUT was clinically relevant^38^. Using the Student’s t-test for paired samples to detect a large effect (Cohen’s d = 0.8) with a power of 80% and a significance level of 0.05, the minimum sample size needed was 15 subjects. Assuming a 30% potential dropout rate because the study follow-up lasted 1 year, at least 20 subjects were needed.

Both eyes were evaluated, and the mean value was computed for analysis. For meibography images, only the data corresponding to one randomly selected eye of each subject was computed for analysis.

Mean values, standard deviations (SD), and 95% confidence intervals (CI) were used to describe quantitative variables (OSDI, CDES-Q, BCVA, LLT, TBUT, corneal staining, MG dropout, and MGYLS score), while median values and interquartile ranges (IQR) were used for ordinal variables (conjunctival hyperaemia, anterior blepharitis grade, and conjunctival staining).

The primary outcome measure to assess conventional LipiFlow efficacy was fluorescein TBUT at 3 months after treatment; however, TBUT was also measured at the 6- and 12-month follow-up visits. The MGYLS score was the secondary outcome measure, and it was assessed at 3-, 6-, and 12 months; however, only the 12-month value was evaluated for statistical significance. The BCVA, as a measure of the safety outcome, was assessed at each visit.

The model assumptions of homoscedasticity, normality, and linearity of effects were checked by a residual analysis. The presence of possible outliers was also checked in the residuals.

For the primary outcome measure, TBUT was compared between the baseline and the 3-month follow-up visit under the normal controlled environment using the Wilcoxon signed-rank test. For the secondary outcome measure, MGYLS was evaluated by linear mixed-effects models that were fitted to assess the effect on the quantitative variables of the LipiFlow treatment over the 12-month study period. For the safety outcome, BCVA was evaluated the same as MGYLS.

To analyse the outcomes of the CDES-Q, the percentage of patients feeling better or the same, or worse after the adverse environmental exposure during the follow-up visits was compared with the percentage before treatment using the McNemar test.

For the quantitative variables (OSDI, CDES-Q-2, BCVA, LLT, TBUT, corneal staining, MG dropout, and MGYLS score) linear mixed-effects models were fitted. In those variables that were measured in both environmental conditions (LLT, TBUT, and corneal staining), the effect of exposure was added to the model. In that case, a likelihood ratio test was used for testing the interaction between time and exposure; *p*-values ≤ 0.1 were considered relevant. To quantify and describe each effect included in the model, least squares means and their differences were estimated. Levels were compared using Tukey's method for the adjustment of multiple comparisons. A residual analysis of all fitted models was performed to check the required assumptions of linear mixed models: linearity, normality of the residuals, homoscedasticity, and no autocorrelation.

For ordinal variables (conjunctival hyperaemia, anterior blepharitis grade, and conjunctival staining), cumulative link mixed models were fitted using the logit function. Parameters were estimated by maximum likelihood using the Laplace approximation. For all fitted models the likelihood ratio test was used to test the interaction effect, in the same terms as for the quantitative variables. The proportional odds assumption was checked for each of the fitted models.

The statistical analyses were performed using the R statistical package (version 4.0.3 (R Core Team 2020)), setting a significance level of 5%.

## Results

### Descriptive analysis

Twenty-five MGD patients were initially recruited for the study; however, four dropped out because of travel and scheduling constraints. Thus, 21 participants (17 females and 4 males) with a mean ± SD age of 59.6 ± 9.4 (range: 30–76) years finished the study. For the recruited patients, the BCVA was 0.02 ± 0.11 log minimum angle of resolution (logMAR), (95% CI: −0.03–0.07); the OSDI was 47.9 ± 26.9 (95% CI: 35.7–60.2); and the MGYLS was 9.9 ± 1.3 (95% CI: 9.3–10.5). The median and IQR value of the anterior blepharitis grade was 2 (1,2).

### Study parameters

#### Outcome measures

Baseline fluorescein TBUT before exposure to the adverse environmental humidity was 4.2 ± 4.1 s, and after LipiFlow treatment was 5.4 ± 7.4 s at 3 months. The change was not significant (*p* = 0.94, Table 1). Neither time nor the adverse environmental humidity had a significant effect (*p* > 0.05 for all comparisons, Table 1). However, after the LipiFlow treatment, there was an improvement of the MGYLS score in the post-adverse environment exposure between the baseline value, 10.3 ± 1.5, and the follow-up visits, (3 months 11.6 ± 1.3, *p* = 0.0008; 6 months 12.4 ± 2.4, *p* = 0.0001, and 12 months 12.2 ± 2.2, *p* = 0.0005, respectively, Table 1).

**Table 1:** Outcomes of Fluorescein TBUT, MGYLS, and BCVA.

Test	Adverse controlled environment	Baseline	3 months	6 months	12 months
TBUT (seconds)	Before	4.2 ± 4.1	5.4 ± 7.4	4.2 ± 3.8	4.3 ± 5.2
		(3.3–6.1)	(2.1–8.8)	(2.4–6.0)	(2.0–6.7)
	After	3.3 ± 1.9	3.1 ± 1.9	3.5 ± 1.5	5.4 ± 7.1
		(2.4–4.6)	(2.3–4.0)	(2.8–4.2)	(2.2–8.7)
MGYLS	After	10.3 ± 1.5	11.6 ± 1.3*	12.4 ± 2.4**	12.2 ± 2.2***
BCVA (logMAR)	Before	0.02 ± 0.09	−0.01 ± 0.11	0.0 ± 0.09	0.0 ± 0.11
		(−0.02–0.06)	(−0.05–0.04)	(−0.04–0.04)	(−0.06–0.05)

TBUT = tear break-up time; MGYLS = Meibomian gland yielding liquid secretion; BCVA = best corrected visual acuity; logMar = log minimum angle of resolution. Values are means ± standard deviation and (95% confidence interval). Comparison with baseline value by post-hoc Tukey´s test: *p = 0.008, **p = 0.0001, ***p = 0.0005.

There was no significant variation in BCVA among the visits (linear mixed model *p* = 0.25, Table 1). The mean value for treatment satisfaction was 8.2 ± 1.4 (range: 5–10), and there were no device-related adverse events reported.

#### Ocular symptoms

The effect of time following LipiFlow treatment on OSDI values was significant (linear mixed model, *p* = 0.008, Table 2). Post-hoc pairwise comparisons revealed that there was a significant (*p* = 0.003) decrease of 14.0 points in OSDI score between baseline and the 12-month follow-up visit.

**Table 2:** Outcomes of Clinical Symptom Questionnaires.

Questionnaire	Controlled environment		Baseline	3 months	6 months	12 months
OSDI	Normal		43.0 ± 23.9	35.5 ± 23.7	35.2 ± 23.2	29.0 ± 22.2*
Mean ± SD (95% CI)			(32.1–53.9)	(24.7–46.3)	(24.6–45.8)	(18.9–39.1)
CDES-Q-1	Adverse	Better & Same	52.4%	66.7%	81.0%	71.4%
(% of patients)		Worse	47.6%	33.3%	19.0%	28.6%
CDES-Q-2 Mean ± SD (95% CI)	Adverse	Better	6.7 ± 1.2	5.7 ± 2.4	6.0 ± 2.5	6.6 ± 2.1
			(5.7, 7.7)	(3.5, 7.9)	(3.9, 8.1)	(4.0, 9.2)
		Worse	4.5 ± 2.8	3.8 ± 1.6	5.5 ± 4.1	4.4 ± 3.0
			(6.4, 2.5)	(5.2, 2.3)	(10.0, 1.1)	(7.8, 1.0)

OSDI = ocular surface disease index (range: 0–100); CDES-Q-1 = change in dry eye symptoms questionnaire 1 (range: better, worse, or the same); CDES-Q-2 = change in dry eye symptoms questionnaire 2 (range: 0–10); CI = confidence interval; SD = standard deviation. *Comparison with baseline value, *p* = 0.003, post-hoc Tukey´s test. No significant differences were found in CDES-Q-1 among visits. OSDI outcomes were measured under normal controlled environment, and CDES-Q outcomes were measured after 2 h of adverse controlled environmental exposure.

After 2 h in the controlled adverse environment, the differences between the percentage in the baseline visit and the remaining visits were not significant in the percentage of MGD patients reporting worsening symptoms after 2 h in the controlled adverse environment (baseline vs 3 months: *p* = 0.375, baseline vs 6 months: *p* = 0.07, baseline vs 12 months: *p* = 0.289, CDESQ-1, Table 2). There were no significant differences in the CDES-Q-2 score (*p* = 0.791, Table 2).

#### Ocular surface signs

There were no significant differences in LLT values under the normal environmental condition at the baseline, 3-, 6-, and 12-month visits (linear mixed model, *p* = 0.29, Table 3). In contrast, the adverse environment exposure significantly decreased the LLT (linear mixed model, *p* < 0.0001). The linear mixed model estimated a 6.5 nm decrease in LLT when patients were exposed to the adverse environmental humidity.

**Table 3:** Outcomes of LLT, conjunctival hyperaemia, anterior blepharitis grade, conjunctival staining, and MG drop out.

Test	Adverse controlled environment	Baseline	3 months	6 months	12 months
LLT (nm)	Before	85.5 ± 11.3	84.2 ± 11.7	81.1 ± 12.0	80.9 ± 14.0
		(79.8–91.5)	(78.7–89.7)	(75.6–86.5)	(74.5–87.3)
	After	78.5 ± 10.4*	77.2 ± 9.8*	75.5 ± 11.9*	74.8 ± 11.1*
		(72.9–84.0)	(72.4–81.9)	(70.1–80.9)	(69.8–79.8)
Conjunctival hyperaemia	Before	1 (1)	1 (1)	1 (1)	1 (1)
	After	1 (1)	2 (1)	1 (1)	1 (1)
Anterior blepharitis	Before	2 (0)	2 (1)	1** (1)	1*** (0)
Conjunctival staining	Before	2 (2)	1 (3)	0^**¶**^ (2)	0^**‖**^ (0)
	After	2 (2)	1 (3)	1 (1)	0 (1)
Upper lid MG dropout (%)	Before	17.7 ± 13.9	20.0 ± 12.3	17.7 ± 12.7	19.1 ± 13.4
		(10.8–24.6)	(13.9–26.0)	(11.9–23.5)	(13.0–25.3)
Lower lid MG dropout (%)		4.3 ± 5.9	7.8 ± 11.8	11.3 ± 18.7	6.4 ± 10.6
		(1.3–7.2)	(2.3–13.3)	(2.8–19.8)	(1.6–22.2)

LLT = lipid layer thickness, MG = Meibomian gland. Values of LLT and MG drop out are means ± standard deviation and (95% confidence interval). Values of conjunctival hyperaemia, anterior blepharitis, and conjunctival staining are median and (interquartile range). *Comparison with before adverse controlled environment *p* < 0.0001 (lineal mixed model). **Comparison with baseline value by post-hoc Tukey´s test, *p* = 0.0005 (cumulative logit mixed model). ***Comparison with baseline value by post-hoc Tukey´s test, *p* < 0.0001 (cumulative logit mixed model). ^¶^Comparison with baseline value by post-hoc Tukey´s test, *p* = 0.01 (cumulative logit mixed model). ^‖^Comparison with baseline value by post-hoc Tukey´s test, *p* = 0.0007 (cumulative logit mixed model).

For conjunctival hyperaemia, neither time nor the adverse environmental humidity had a significant affect (*p* > 0.05 for all comparisons, Table 3).

The anterior blepharitis grade also varied among visits (*p* < 0.0001, Table 3). The scores corresponding to the 6- and 12-month visits were lower in comparison with the baseline visit (*p* = 0.01 and *p* = 0.0007, respectively, Table 3). Specifically, the odds-ratio for increasing one unit in anterior blepharitis grade during the 6- and 12-month visits were 0.08 (95% CI: 0.01–0.58) and 0.008 (95% CI: 0.0006–0.14), respectively.

**Figure 2:**
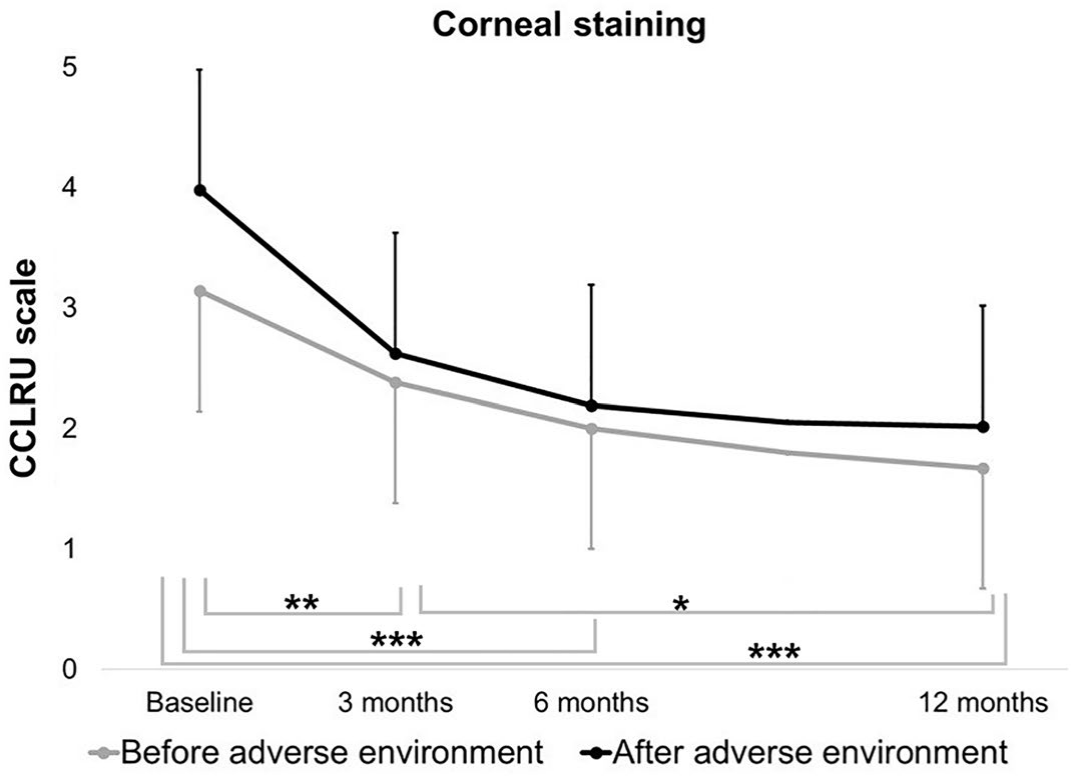
Fluorescein corneal staining. Scores based on the Cornea and Contact Lens Research Unit (CCLRU) scale were determined at baseline and at the 3-, 6-, and 12-month follow-up visits, before and after the exposure to the adverse controlled environment. Mean values and confidence interval error bars are represented. Grey bars represent comparisons between visits during exposure to the normal environment. *p = 0.0314, **p = 0.0001, ***p < 0.0001. The adverse environmental exposure had a significant (*p* = 0.0165) negative effect on corneal staining regardless of the visit. The interaction between the effect of the time and the conditions was not significant (*p* = 0.49).

For fluorescein corneal staining, the interaction between the effect of the treatment over time and the adverse environment was not significant (*p* = 0.49). However, both time (*p* < 0.0001) and environmental conditions (*p* = 0.0165) have a significant effect separately, according to the lineal mixed-effects model. Corneal staining decreased significantly at each visit (3 months: *p* = 0.0001, 6 months: *p* < 0.0001, 12 months: *p* < 0.0001, Fig. 2). In addition, the decrease between the 3- and 12-month visits was significant (*p* = 0.0314, Fig. 2). Regarding the adverse environment, the model showed that fluorescein corneal staining increased at all visits after the exposure (*p* = 0.0165).

Conjunctival staining was decreased at the 6- and 12-month follow-up visits (*p* = 0.0005 and *p* < 0.0001 respectively, Table 3) compared to the baseline visit. The odds-ratio for increasing one unit of the conjunctival staining score during the 6- and 12-month visits were 0.22 (95% CI: 0.09–0.51) and 0.06 (95% CI: 0.02–0.17), respectively. Thus, the likelihood for decreasing one unit of the conjunctival staining score during the 6- and 12-month visits were 4.54 and 16.66 times, respectively. In contrast, there was no effect of the adverse environmental humidity on conjunctival staining compared to the baseline visit (*p* = 0.54).

MG dropout for the upper and lower lids did not change among the visits (*p* = 0.32 and *p* = 0.24, respectively, Table 3).

## Discussion

LipiFlow treatment has emerged as a common clinical procedure for the management of MGD^12,13,15^, especially for people who spend most of their time under indoor environmental conditions that typically can include high temperature, low relative humidity, and/or air drafts. These conditions can negatively affect the tear film and trigger DE exacerbation in normal and DE patients as well as those who wear contact lenses^28,43^. In addition, the outdoor environment also plays an important role regarding DE^27,50^. Thus, we aimed to assess the efficacy of LipiFlow therapy not only under a normal environment but also under a controlled environment to determine if this therapy also provides a further prophylactic effect against adverse environments.

In the present study, the safety outcome measure selected was BCVA. As expected, there were no significant changes among baseline and follow-up visits, and there were no device-related adverse events. Thus, LipiFlow therapy can be considered to be a safe MGD treatment if the instrument manufacturer’s recommendations are followed.

To carry out the study, two different environmental conditions were selected based on studies previously performed by our research group using a validated exposure model within an environmental chamber^32,38,51^. In addition, the American Society of Heating, Refrigerating and Air-Conditioning Engineers (ASHRAE, USA) recommends that the indoor relative humidity be maintained below 60%^52^, and the comfort range for most people is between 40 and 60%^53^. Therefore, to assess the efficacy of LipiFlow therapy, we selected a controlled environment with 50% relative humidity. ASHRAE also recommends an indoor temperature range between 20.5° and 24 °C in the winter for a 50% indoor relative humidity. Thus, we selected a temperature of 23 °C for this study. For the adverse environment, the temperature was also 23 °C, and a 10% relative humidity was selected because low relative humidity values are often present during the winter season within office buildings and dwellings situated in northern climates^54^.

Our primary expectation was that LipiFlow treatment would increase TBUT. However, at neither 3 months nor any of the other follow-up visits was TBUT significantly increased over the baseline value for both conditions (normal and adverse). The effect of LipiFlow treatment on TBUT values is usually short-term. Previous authors have observed improvements of TBUT values for 4 weeks after LipiFlow therapy^10,21^. However, Zhao et al.^21^ found that the 4-week improvement was not sustained at 3 months after treatment. Greiner et al.^4,11^ reported that TBUT was increased at 1 month after LipiFlow treatment, though this improvement was not sustained at the 9- and 12-months post-treatment visits. Satjawatcharaphong et al.^55^ did not find any significant change in TBUT values during a mean follow-up period of 52 days after LipiFlow treatment. Likewise, Yeo et al.^22^ did not find significant changes in TBUT at 3-months post-treatment. Thus, while TBUT values may increase during the first weeks after LipiFlow treatment, the improvement does not appear to be sustained after a few months. In our study, the first follow-up visit was scheduled 3 months after treatment, and this is likely to be the reason for not observing significant changes in TBUT after LipiFlow therapy.

Our secondary expectation was that the MGYLS score would increase following LipiFlow treatment. In fact, it increased significantly at the 3-month follow-up visit, and the effect was sustained through the 12-month visit. Similar to the present study, Greiner et al.^11^ observed a MGYLS score improvement at 1-year post-treatment. Thus, LipiFlow therapy can produce a long-term effect on MG secretion that is associated with rapid improvement after treatment and is sustained over a long follow-up period.

After the improvement of the MG secretion, an increase in the LLT might have been expected. However, there were no differences between visits. This finding agrees with other authors that did not find differences after 1, 3, and 12 months^19,21^. Conversely, a retrospective study reported an improvement in LLT 4 and 12 weeks^14^ after LipiFlow treatment, and Finis et al.^19,56^ found an increase in LLT after 6 months, but not after 3 months. The reason for observing different results in the literature might be associated with the baseline LLT values. In the present study, the mean baseline LLT was high, near 70 nm, whereas Finis et al. had an inclusion criterion of LLT ≤ 60 nm^19,56^. Our results are similar to those of Blackie et al.^12^, who had an inclusion criterion of LLT ≤ 80 nm. Therefore, the likelihood of finding an improvement in the LLT may be higher when the LLT is thinner.

In the present study, anterior blepharitis grading was improved at the 6- and 12-month follow-up visits. It is likely that LipiFlow therapy decreases the cystic dilation of the ducts associated with the stasis of meibum typically observed in MGD. This reduction in the dilation of the ductal system can result in reduced clinical signs of eyelid inflammation. To the best of our knowledge, this is the first time that an improvement in anterior blepharitis grade after LipiFlow treatment has been reported. This outcome has been included before in only other one study as the safety measure^14^, but it is commonly assessed when evaluating other MGD treatments such as warm compresses, lid hygiene, and Meibomian gland probing^12^.

Regarding the ocular surface integrity, we found that LipiFlow treatment decreased corneal fluorescein staining scores during all follow-up visits, before and after the adverse exposure, and Lissamine green conjunctival staining scores were decreased at the 6- and 12-months visits before the adverse exposure. Previous authors have also reported an improvement of corneal staining at 1 and 3 months after treatment^10,21,23^. In contrast, Finis et al.^19^, using the Oxford scale, evaluated the combined corneal and conjunctival staining. They observed an improvement 1 month after treatment that returned to baseline levels at the 3-month follow-up visit. Previous authors have used different validated methods to assess corneal and conjunctival staining after LipiFlow treatment. Thus, from a statistical viewpoint, using different grading scores might have contributed to the differing results among the studies. However, regardless of severity, the chronic presence of ocular surface staining in DE patients is always related to inflammation^57^, a condition that should be resolved or at least ameliorated. MGD patients suffer mainly from evaporative-subtype dry eyes. Thus, the severity of the ocular surface integrity scores (i.e., corneal staining) are lower than in patients suffering from the aqueous deficient subtype of DE alone. Consequently, the surface integrity scores after any treatment (i.e., LipiFlow) are also expected to be lower.

After LipiFlow treatment, we found a noteworthy decrease in the subjective OSDI scores at 12 months. The improvement in subjective outcomes has also been reported in most of the previous studies assessing LipiFlow therapy^8^. Consequently, LipiFlow therapy provides objective and subjective improvements of the ocular surface in MGD patients. In this study, the participants’ mean satisfaction rating of the treatment after completing each follow-up visit was 8.2 (range: 0–10). Therefore, LipiFlow therapy seems to be a reasonable clinical procedure from the MG patient´s perspective.

While several studies have already proven the objective and subjective efficacy of LipiFlow treatment, the present study is the first to assess MGD patients who were always examined under the same environmental conditions. Likewise, it has not been previously addressed if the post-treatment improvement commonly reported was also consistent enough to protect MG patients from DE exacerbations under an adverse environment. We found that exposing MG patients to the adverse environmental humidity produced a negative effect on LLT and on corneal staining. This worsening of the ocular surface has been previously observed, not only in mild to moderate DE patients while exposed to desiccating stress, but also in normal subjects and contact lens wearers^27,29,32^. Based on the corneal staining results, a decrease of the worsening after exposure to the adverse environment could be expected at 3- and 6-months visits after LipiFlow treatment. However, the differences in the worsening of corneal staining among visits did not reach statistical significance, as assessed by the interaction between time and adverse environment.

Regarding the subjective outcomes, CDES-Q-1 results showed that the percentage of patients reporting worsening of symptoms after 2 h of adverse environment during baseline visit was higher, 47.6%, in comparison with the percentage observed during the 3-, 6- and 12-month follow-up visits, i.e., 33.3%, 19.0%, and 28.6%, respectively. However, this improvement, like that of the corneal staining, was not statistically significant, even at the 6-month visit when the differences were highest. The absence of significant findings can be attributed to the small sample size, which was calculated based on TBUT, the primary outcome measure. Thus, larger study population samples might show further evidence of the possible prophylactic effect of LipiFlow therapy against adverse environments.

An important finding of this study is that neither the signs nor symptoms of patients exposed to lower humidity after LipiFlow treatment worsened as much as they did during the baseline visit after the adverse environment. The corneal staining (CCLRU) scores observed during the 6- and 12-month visits after the adverse exposure tended to be healthier than those of the baseline visit prior to undergoing the adverse exposure, although the interaction between the effect of the time and the environmental conditions was not significant. Likewise, the percentage of patients indicating that were better or the same (CDES-Q-1) after the adverse exposure was 52.4%, 81.0%, and 71.4% at the baseline, the 6- and 12-month visits. There were no significant differences in this percentage.

Several studies performed by our research group have shown that subjects with or without dry eye worsened under controlled adverse conditions^28,51,58^; therefore, a prophylactic effect of LipiFlow treatment is a reasonable explanation of these findings, although more studies are needed to confirm these outcomes.

Patients with chronic DE often suffer from episodic flares of inflammation (DE exacerbation) that can be triggered by environmental stresses^59^. Previous authors have shown the potential of several DE treatments (e.g., anti-inflammatory therapies) to also inhibit the activation of acute flare provoked by a controlled adverse environment^36–38,60^. Therefore, to ameliorate flares in MGD patients suffering from DE, LipiFlow therapy could also be recommended in the future as an adjuvant treatment in combination with other DE therapies with prophylactic benefits against adverse environment exposures.

The main limitation of the present study is the absence of a control group undergoing other MGD therapy or no therapy. Nonetheless, Pang et al.^61^ have performed a meta-analysis of randomized controlled trials comparing the efficacy of LipiFlow and warm compress treatment for DE management. They concluded that a single session of LipiFlow was highly efficacious for treating signs and symptoms of DE. A second limitation of this study was that MGD patients were not masked, thus the placebo effect cannot be excluded, and the actual improvement of symptoms could be lower than reported by patients.

## Conclusions

In conclusion, the present study showed that a single session of LipiFlow treatment can improve objective and subjective outcomes of the lacrimal functional unit in MGD patients assessed under controlled environmental conditions. Moreover, the benefits of the therapy are sustained for at least one-year post-treatment. However, for the hypothesis that LipiFlow treatment could be provided as an adjuvant therapy to help MGD patients avoid acute flares when exposed to an adverse environmental humidity, larger sample size studies must be performed to confirm the possible prophylactic action.

## Data Availability

The datasets analysed during the current study are available from the corresponding author on reasonable request.

## Abbreviations

ASHRAE: American society of heating, refrigerating and air-conditioning engineers
BCVA: Best corrected visual acuity
CCLRU: Cornea and contact lens research unit
CDES-Q: Change in dry eye symptoms questionnaire
CI: Confidence interval
DE: Dry eye disease
ETDRS: Early treatment diabetic retinopathy study
IQR: Interquartile ranges
LLT: Lipid layer thickness
logMAR: Log minimum angle of resolution
MG: Meibomian gland
MGD: Meibomian gland dysfunction
MGYLS: Meibomian gland yielding liquid secretion
OSDI: Ocular surface disease index
SD: Standard deviation
TBUT: Tear break-up time
